# An Abbreviated Diagnostic Maneuver for Posterior Benign Positional Paroxysmal Vertigo

**DOI:** 10.3389/fneur.2016.00115

**Published:** 2016-07-18

**Authors:** Pia Michael, Carolina Estibaliz Oliva, Marcia Nuñez, Cristian Barraza, Juan Pablo Faúndez, Hayo A. Breinbauer

**Affiliations:** ^1^Otolaryngology Department, San Juan de Dios Hospital, University of Chile, Santiago, Chile; ^2^Clínica Alemana de Santiago, Facultad de Medicina Clínica Alemana, Universidad del Desarrollo, Santiago, Chile

**Keywords:** benign paroxysmal positional vertigo, vestibular function test, Dix–Hallpike maneuver, diagnostic test assessment, dizziness

## Abstract

**Introduction:**

Benign paroxysmal positional vertigo (BPPV) secondary to canalolithiasis of the posterior semicircular canal is perhaps the most frequent cause of vertigo and dizziness. One of its properties is a high response rate to canalith repositioning maneuvers. However, delays in the diagnosis and treatment of this entity can range from days to years, depending on the setting. Here, we present an abbreviated variation of the Dix–Hallpike maneuver, which can be used to diagnose this disease. It is similar to the standard maneuver but can be performed without an examination bed/table and requires only a backed chair (a difference that we feel is very important in settings where a clinical bed/table is not readily available).

**Methods:**

A diagnostic assessment study was conducted in 163 patients who presented with vertigo or dizziness.

**Results:**

The abbreviated test had fairly good sensitivity (80%) and high specificity (95%) for diagnosing posterior BPPV.

**Discussion:**

This new diagnostic maneuver may serve as a screening procedure for quickly identifying this pathology. This will allow patients to be more directly treated, without requiring unnecessary referrals or full vestibular testing, and will be especially useful in primary care settings or heavily overloaded otolaryngology or neurology departments.

## Introduction

In the following study, we describe the development and the results of initial testing of a shortened version of the standard Dix–Hallpike (sDH) maneuver, which we have called Abbreviated Posterior Canalolithiasis Chair-based Assessment Maneuver (APCCAM). This version is useful for diagnosing the posterior canalolithiasis variant of benign paroxysmal positional vertigo (BPPV). We propose that this diagnostic tool has practical value, particularly for physicians who are not specialized in vestibular disorders because it will help them to easily identify a large majority of simple BPPV cases, thus allowing instant treatment for these patients and avoiding unnecessary referrals. This reliable and easy-to-perform diagnostic maneuver does not require an examination bed or table.

Benign paroxysmal positional vertigo is the most frequent cause of vertigo, with a lifetime prevalence of 2.4% and an estimated year-prevalence of greater than 9% in adults older than 60 years. BPPV represents 17–25% of all patients who present with vertigo or dizziness in specialized units (1–3). The prevalence of BPPV increases with age and is associated with an increased risk of falling, which is a major health issue in the elderly (4, 5). Originally described by Robert Barany in 1921 (6) and properly defined by Margaret Dix and Charles Hallpike in 1952 (7), BPPV is clinically characterized by brief spells of positional vertigo or dizziness (these symptoms are triggered by a change in the position of the head in space relative to gravity) that can last from a few seconds to a few minutes (8, 9).

Benign paroxysmal positional vertigo represents a common clinical entity that is encountered not only by specialists in neuro-otology and balance disorders but also by non-specialized otolaryngologists, neurologists, or geriatricians and general practitioners in primary care or emergency departments, among many other settings, in routine clinical practice (10–12). It is widely accepted that BPPV is caused by the dislodgement of otoconia from the otolith macula (8, 12). These particles then float until they become trapped within a semicircular canal (canalolithiasis) or attached to its cupula (cupulolithiasis). Then, after a change in head position in the plane of the affected canal, gravity induces the trapped otoconia to move, resulting in abnormal endolymph flow and the subsequent deflection of the cupula in cases of canalolithiasis or direct cupular deflection in cases of cupulolithiasis. In both scenarios, the vestibular afferents from the affected canal are modulated (stimulated or inhibited) in an abnormal and augmented fashion, particularly in comparison to the “paired canal” in the contralateral ear, which lacks the “extra weight” of the dislodged otoconia required to react normally to head movements. The computation of this asymmetry at the vestibular nuclei triggers not only vertigo or dizziness but also a specific type of nystagmus that depends on the canal that is affected by the disease. All three semicircular canals can be afflicted by this condition (11–13).

Among all possible BPPV variants, canalolithiasis of the posterior semicircular canal (pc-BPPV) is by far the most frequently encountered, representing 80–95% of all BPPV cases. It is therefore the single most common specific cause of vertigo (12–15).

**Figure 1:**
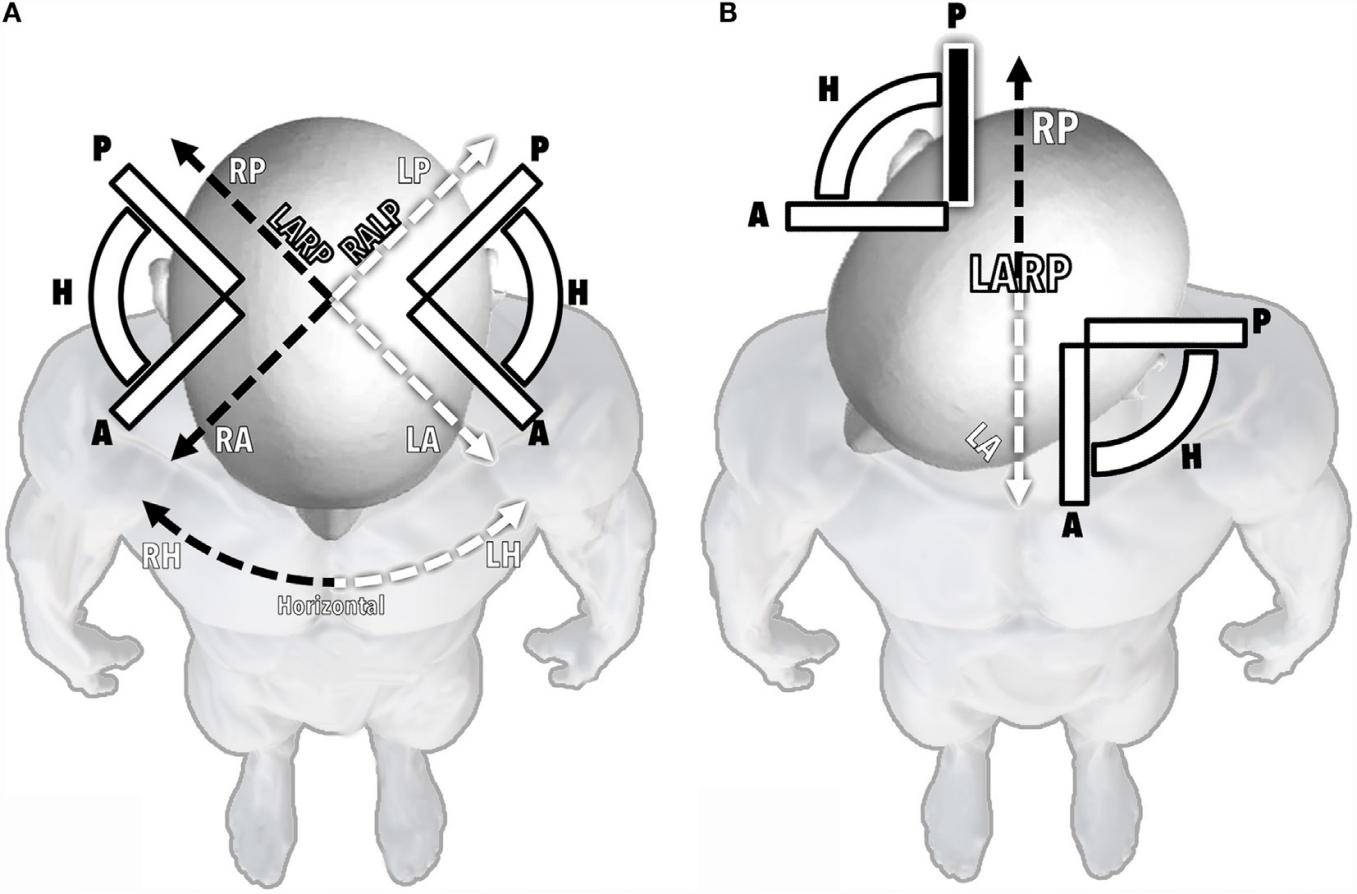
**The disposition of semicircular canals**. The alignment of all six canals is represented as seen from above a patient’s head. **(A)** Head in a neutral position. **(B)** Head turned 45° to the right, thus aligning the LARP plane and the left anterior and right posterior canals with the sagittal plane of the body below the neck. A, anterior canal; P, posterior canal; H, horizontal canal; RA, RP, and RH, right anterior, posterior, and horizontal canals, respectively; LA, LP, and LH, left anterior, posterior, and horizontal canals; LARP, left anterior/right posterior plane; RALP, right anterior/left posterior plane.

The posterior canals share their plane of rotation with the anterior canal of the contralateral ear. These planes were therefore named “RALP” (right anterior/left posterior) and “LARP” (left anterior/right posterior). For practical reasons, the spatial disposition of these planes is generally simplified and visualized as having a 45° deviation from the sagittal plane of the head (Figure 1A). If the head is turned 45° to the right, the LARP plane (and therefore the right posterior canal) becomes aligned to the sagittal plane of the rest of the body below the neck (Figure 1B).

**Figure 2:**
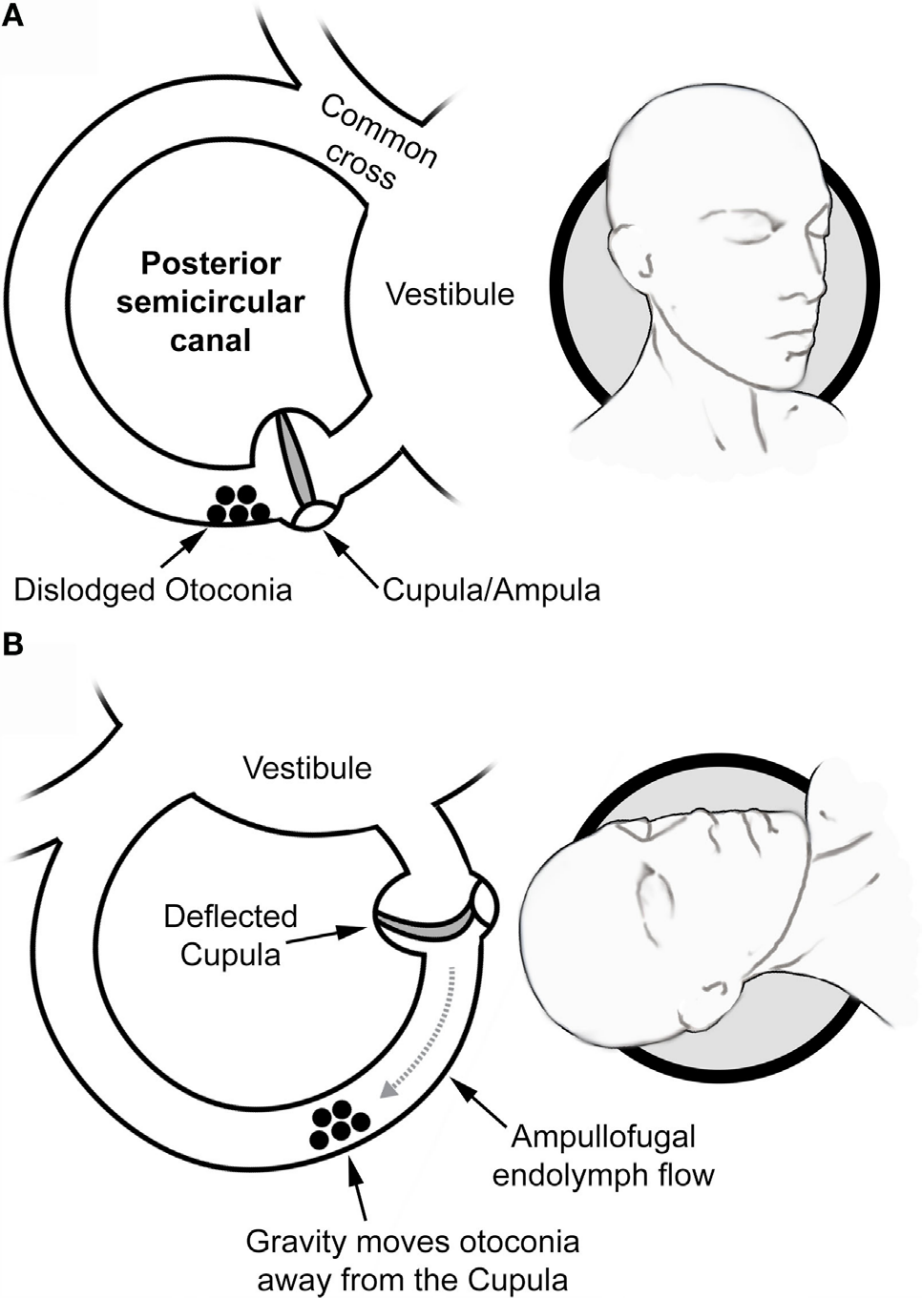
**Mechanisms in canalolithiasis**. **(A)** With the head in an upright position, a dislodged otoconia is shown in a pc-BPPV patient to be resting within the posterior canal near the cupula region. **(B)** If the head is first turned 45° toward the affected side and then pulled backward in the plane of the affected canal, gravity induces the otoconia to move downward and thus away from the cupula. This generates ampullofugal endolymph flow, which deflects the cupula away from the vestibule. This triggers an excitatory vestibular afferent signal, which leads to the characteristic nystagmus that is detailed in Figure 3.

This arrangement is key to the classic sDH maneuver, in which the patient, while in a sitting position with the head turned 45° to the side, is quickly laid back until a supine head-hanging position is reached (7, 16). Then, the head is rotated and translated in either the RALP or the LARP plane. If loose otoconia are present within the posterior canal being tested, gravity moves them away from the cupula, which generates ampullofugal endolymph flow (Figure 2).

**Figure 3:**
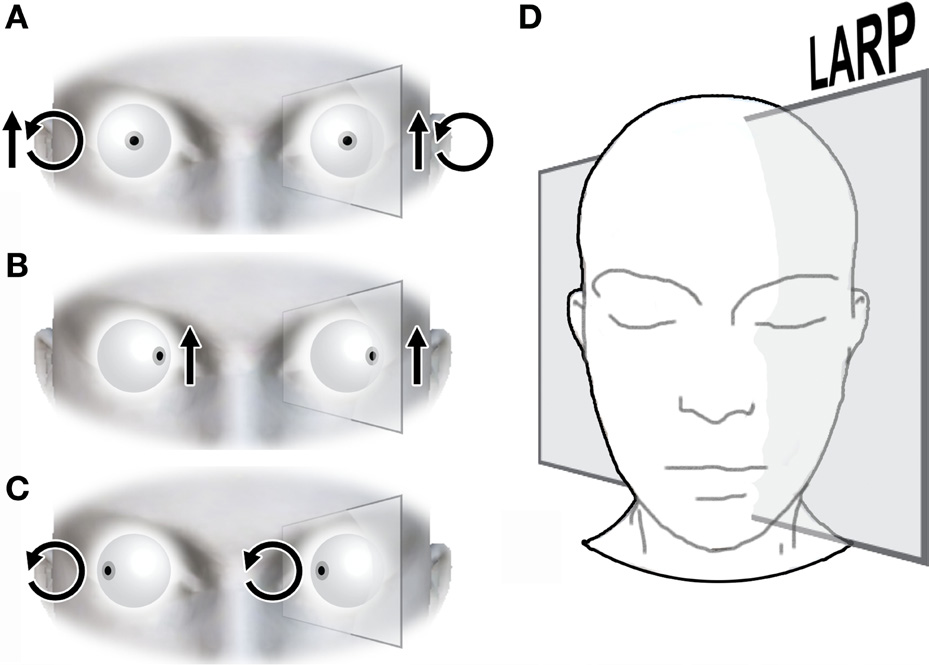
**Nystagmus characteristic of pc-BPPV**. **(A)** A right pc-BPPV will present, after a Dix–Hallpike maneuver, with nystagmus accompanied by a quick phase that beats upward and rotates toward the affected ear (e.g., the upper pole of the eye rotates toward the right side). The eye rotates three-dimensionally in the LARP plane. From a frontal perspective, this is perceived as a mixed vertical and torsional nystagmus. **(B)** If the patient is asked to look to the left, thus aligning his gaze with the LARP plane, the pupil will beat upward in this plane, isolating the vertical component. **(C)** When the patient directs his gaze rightward, it becomes perpendicular to the LARP plane. In this position, the pupil is near the axis of rotation, therefore isolating the torsional component. **(D)** The eyes rotate on an axis parallel to the LARP plane.

According to Ewald’s laws, this triggers the eyes to move in the same plane as the plane of the canal being stimulated (17). For example, if the right posterior canal is affected, the result is a nystagmus with both torsional and vertical components (Figure 3).

These eye movements can be better evaluated by shifting the gaze sideways. In the case of an affected right posterior canal, when a patient is asked to move his eyes to the left, he aligns his gaze with the LARP plane (Figure 3B). In this setting, only the vertical nystagmus component will be visualized (as a slow phase pulling the eyes downward in this diagonal plane and a visible quick phase directing them upward). In contrast, if the patient shifts his gaze to the right, thus bringing his gaze perpendicular to the LARP plane, the torsional component is isolated, and it initiates a slow phase that rotates the eyes clockwise and a quick phase that rotates eyes counterclockwise (Figure 3C).

Usually, patients are examined while their gaze is neutral and forward. In this setting, both components are combined, and this creates the characteristic nystagmus of pc-BPPV, with the vertical component beating upwards (toward the forehead) and the torsional component beating the upper pole of the eye toward the affected ear (8) (Figure 3A).

Observing this nystagmus after performing an sDH in a patient presented with recurrent but brief (less than 1 min long) attacks of positional vertigo or dizziness that were provoked by lying down or rolling over into a supine position (among other movements) is the main diagnostic criteria for pc-BPPV (8).

Another key feature of pc-BPPV is its excellent response to treatment (14, 18), in that the immediate disappearance of the positional nystagmus and other symptoms after performing canalith repositioning procedures (CRPs) is viewed as strongly supporting the diagnosis (8). Both the Semont and, in particular, the Epley CRPs have been shown to reliably resolve most pc-BPPV cases, even when applied as a single procedure that was only a few minutes in duration (14, 19–22).

A “subjective” form of pc-BPPV has also been described to involve positional vertigo symptoms that are triggered after an sDH, but without a nystagmus (23). It has been proposed that in these cases, the amount of loose otoconia is sufficient to produce symptoms but is insufficient to trigger abnormal eye movement responses (8, 23, 24). In these cases, improvement in symptomatology after CRPs supports both the idea that subjective pc-BPPV is a valid entity and the accuracy of diagnosis on a patient to patient basis (24).

Overall, most pc-BPPV cases can be easily diagnosed and treated using an sDH followed by CRPs within a single medical visit (3, 12, 13, 15, 18, 24).

However, many patients with this condition wait several months or even years to have it properly diagnosed and treated (25, 26). Different studies have reported that the *average* time from symptom onset to diagnosis can be 19–70 months and require more than eight visits to a medical center before a diagnosis of BPPV is achieved (27, 28). Furthermore, it is estimated that 10% of elderly patients who suffer from dizziness or unsteadiness have unrecognized BPPV even though they are routinely assessed by medical providers (4, 29).

The delay in diagnosis and treatment of BPPV has been attributed to many different causes. There is a tendency in some settings to refer all cases of vertigo to otolaryngology, neurology, or vertigo-specialized units, which overload these specialties (1, 26, 30). Additionally, even in simple and uncomplicated BPPV cases, unnecessary imaging and vestibular tests are frequently ordered (26). However, for non-specialized neurologists, otolaryngologists, or general practitioners, performing a simple sDH may be a greater challenge than referring the patient for full vestibular testing. In many settings, particularly in overloaded primary care facilities and even many otolaryngology practices, the underlying reason for this behavior is that the practitioner does not have easy access to an examination table or bed to perform testing.

Given the above information, we believe that developing an abbreviated and easy-to-teach diagnostic maneuver that requires minimal infrastructure to be performed and that focuses solely on pc-BPPV as the single most common cause of vertigo may lead to a screening-like procedure for this entity, and this may lead to an instant diagnosis–treatment algorithm that will ideally decrease unnecessary referrals and patient care delays.

## Materials and Methods

First, consistent with the principles of the sDH, we developed a simplified maneuver that we have identified with the acronym APCCAM. We then conducted a diagnostic test to evaluate the effectiveness of this new maneuver in which we used the sDH as the gold standard.

### Description of APCCAM

#### Requirements

A backed chair is necessary to perform an APCCAM. We intentionally describe the test as being performed without Frenzel glasses or a video-oculography device. These devices are of extraordinary value when assessing pathological eye movements and vestibular disorders, but we intended to the APCCAM to require a minimum of material aids to support its widespread, non-specialized use.

#### Performing the APCCAM

##### Step 1

**Figure 4:**
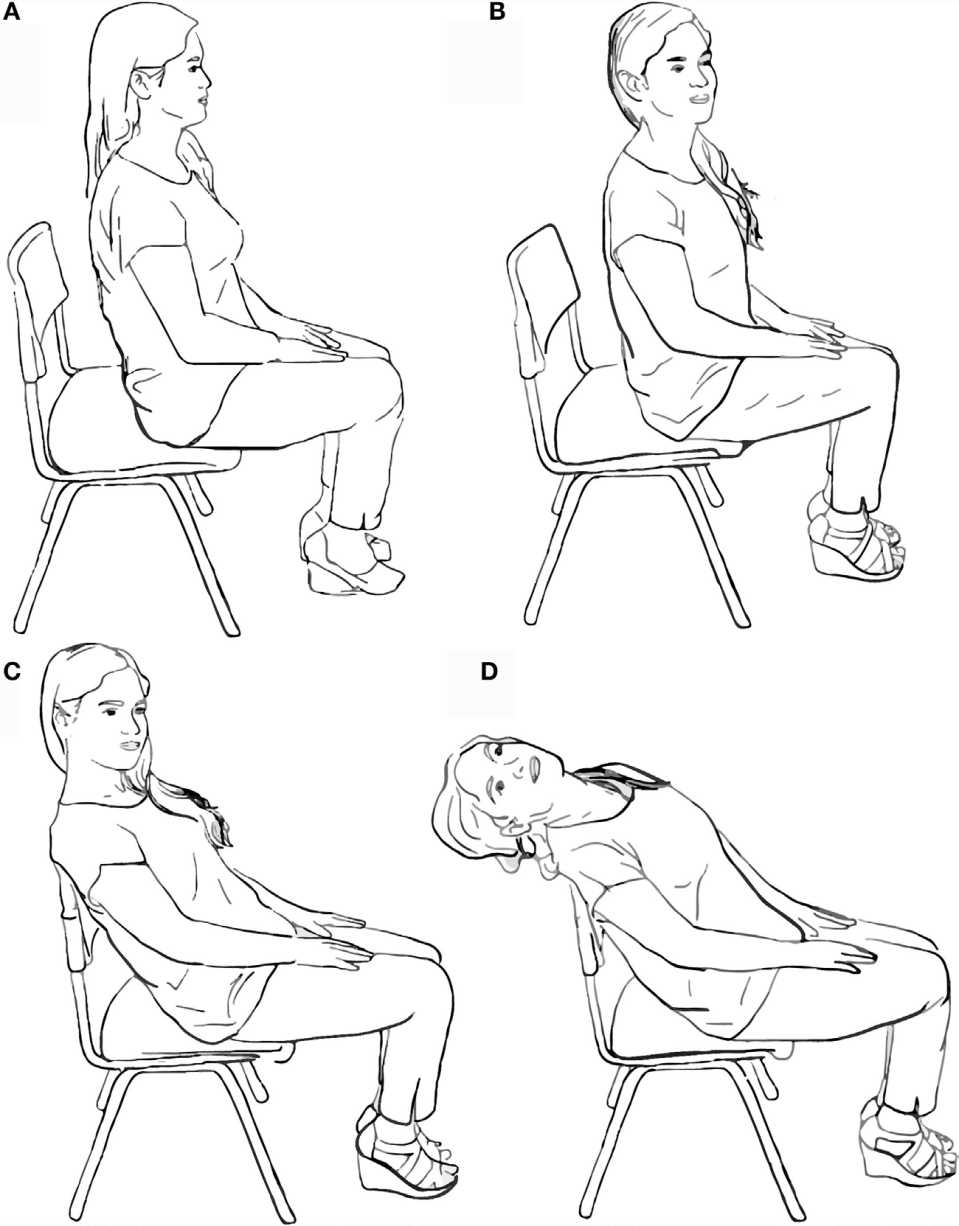
**Performing the mini Dix–Hallpike maneuver**. **(A)** First, the patient is asked to sit on the front edge of a backed chair. **(B)** The patient’s head is then turned 45° toward the side being examined. **(C)** The patient is pulled backward into a resting position against the back of the chair. **(D)** The patient’s head is then pulled backward into a hanging position.

The patient is asked to sit on the front edge of the backed chair (Figure 4A). This step is critical for securing a wider range of neck movement.

##### Step 2

The patient is asked to turn his/her head 45° sideways, toward the side being assessed. Figure 4B shows a patient turning her head to the right, which aligns her LARP plane with the sagittal plane of the rest of her body (see also Figure 1B). As previously explained, this is key to assessing the posterior canal of the right ear.

##### Step 3

The patient is asked to lay back until they are resting against the back of the chair (Figure 4C). As in step 1, this allows a wider range of neck movement.

##### Step 4

The professional performing the APCCAM guides the head of the patient as far back as possible (Figure 4D). This resembles the main action performed in the sDH and is responsible for the main rotation and translation of the posterior canal relative to gravity. As previously explained, this step induces the movement of dislodged otoconia, which triggers nystagmus and symptomatology (see also Figure 3). At this point, the patient’s eyes should be observed for nystagmus, and the patient should be questioned regarding symptoms.

##### Notes

Steps 3 and 4 should be carried out as seamlessly and as quickly as possible. As with the final position in the sDH, step 4 should be maintained for at least 20 s because of the known latency of nystagmus and the symptomatology of pc-BPPV (7, 12, 23). Steps 2–4 should be repeated to assess the posterior canal of the contralateral ear. Additionally, similar to the sDH, the professional conducting the APCCAM should take the patient’s head firmly in his/her hand and guide every step of the maneuver. The presence of the examiner is purposely omitted in Figure 4 to more clearly demonstrate the head and body position of the patient at every step.

#### Positive Findings (Diagnostic Criteria)

We decided to evaluate two independent positive results as potential diagnostic criteria supporting pc-BPPV:

(a) The presence of pc-BPPV-characteristic nystagmus (8, 12) (Figure 3).
(b) Symptoms of unilateral positional vertigo or positional dizziness (8) that were triggered while using the APCCAM.

We acknowledge the possibility of bilateral pc-BPPV. However, unilateral pc-BPPV is much more common, and we therefore focused on the latter scenario while developing the APCCAM. We surmised that if a patient reports symptoms that are clearly localized on one side and not on the other that a diagnosis of pc-BPPV is more likely than cases in which the patient feels “something” equally on both sides (which can be easily confused with orthostatic phenomena). This may prove to be a limitation of this tool over time, but at this stage, we feel confident in making unilaterality a criterion for a positive diagnosis.

### Testing of the APCCAM

A diagnostic test study was conducted. Four otolaryngology residents and one member of the staff were trained to execute the APCCAM. From January to February 2016, patients presenting with vertigo or dizziness to one of these five physicians at the Otolaryngology Department of the Hospital San Juan de Dios in Santiago de Chile were invited to participate. The rest of the department’s physicians were asked to refer patients presenting with histories characteristic of BPPV to this assessing group. The exclusion criteria included severe neck, ophthalmological, or neurological pathology, previously known vestibular disorders, or the presence of spontaneous nystagmus.

When the APCCAM was performed, we registered whether it triggered a nystagmus or other symptomatology. An appointment for vestibular testing (including complete positional testing) was then scheduled. As a result of scheduling overloads in our department, this appointment did not occur on the same day as the initial medical assessment. We note this to acknowledge that the known elements of BPPV fatigability with positional testing were ruled out.

The sDH was performed at this second stage with the support of video-oculography. For the purposes of this study, only the triggering of nystagmus was considered to be a positive (abnormal) sDH result. If abnormal findings other than those associated with BPPV were found, the patient was scheduled for medical reassessment.

If pc-BPPV was detected at this stage, the Epley CRP was immediately performed, and the patient was scheduled for weekly follow-ups until the positional vertigo and nystagmus disappeared.

Using the sDH results as the gold standard diagnostic test, the APCCAM results (considering nystagmus and unilateral symptoms to be positive/abnormal findings) were analyzed in terms of sensitivity, specificity, and receiver operating characteristic (ROC) curves.

## Results

A total of 163 patients participated in the study. They had an average age of 54.3 (SD 19.5) years old, ranging from 18- to 92-year-olds. In terms of gender, 112 patients (68.7%) were females. The average delay between symptom onset and assessment was 16.6 (SD 17.44) months, ranging from 1 week to 72 months (6 years). No patients complained of pain or other specific forms of discomfort during the APCCAM.

Forty-five patients (27.6%) presented with a typical nystagmus (Figure 3) indicating unilateral pc-BPPV during the APCCAM. Furthermore, 31 patients (18.4%) stated that unilateral symptoms (vertigo or dizziness) were experienced during the APCCAM, but that no symptoms were triggered when the APCCAM was performed for the contralateral ear. Overall, 76 patients (46.6%) presented with either nystagmus or unilateral symptoms while performing the APCCAM.

Ninety-one patients (55.8%) presented with nystagmus characteristic of unilateral pc-BPPV while performing the sDH, which was observed using video-oculography. All sDH-positive patients experienced a resolution of their symptoms after one to three sessions of the Epley CRP.

None of the 72 patients who displayed no signs of pc-BPPV with the sDH presented with nystagmus while performing the APCCAM, and only one patient noted unilateral symptoms.

No patients presented with nystagmus or phenomena, suggesting central positional nystagmus.

These results are summarized in Table 1. When we considered only the presence of nystagmus (treating other symptoms as irrelevant) as a criterion for pc-BPPV, the APCCAM had a sensitivity of 49.5% [95% confidence interval (CI) between 38.8 and 60.1%] and a specificity of 100% (95% CI between 95 and 100%). When the presence of unilaterally triggered symptoms was also viewed as a positive/abnormal criterion, APCCAM sensitivity was 80.2% (95% CI between 70.6 and 87.8%), and specificity was 95.8% (95% CI between 88.3 and 99.1%).

**Table 1:** **The sensitivity and specificity of the APCCAM**.

	APCCAM triggered nystagmus	APCCAM triggered unilateral symptoms but no nystagmus	APCCAM triggered no symptoms or nystagmus	Total
sDH positive^a^	45	30	16	91
sDH normal	0	1	71	72
Total	45	31	87	163
Sensitivity^b^ (95% confidence interval)	49.5% (38.8–60.1%)	80.2% (70.6–87.8%)		
Specificity^b^ (95% confidence interval)	100% (95–100%)	95.8% (88.3–99.1%)		

*The main results in each group are summarized*.

*^a^An sDH was considered positive or abnormal only in cases when nystagmus characteristic of pc-BPPV was observed via video-oculography*.

*^b^Sensitivity and specificity are expressed as percentages and 95% confidence intervals*.

**Figure 5:**
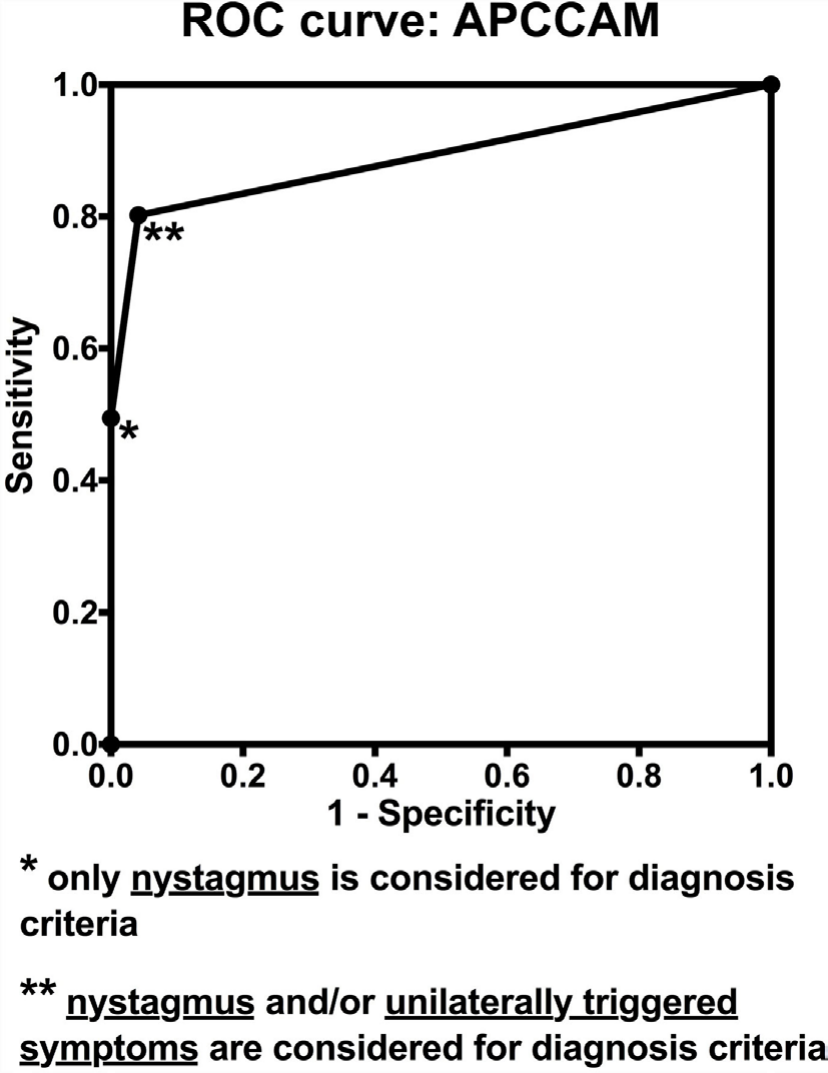
**ROC curves for the APCCAM**.

The ROC curve analysis (considering nystagmus only and nystagmus or triggered symptoms as two levels or scores for diagnosis) of these results is shown in Figure 5, in which we obtained an area under the curve of 0.89 with a 95% CI between 0.84 and 0.94 (*p* < 0.0001).

## Discussion

The APCCAM is essentially very similar to the classic sDH. The sequence of positions, translations, and rotations that are performed to move the head in space relative to gravity follow the same principles as the sDH. The only difference is the replacement of the examination bed/table with a backed chair, an implement that is thought to be more readily accessible for more widespread use. This compromises the range, speed, and fluidness of the traditional maneuver. Moreover, and further diminishing the theoretical reliability of this canalolithiasis diagnostic maneuver, we tested the APCCAM without using Frenzel glasses or video-oculography.

The APCCAM showed fair sensitivity (80%) and high specificity (96%) when not only triggered nystagmus but also unilateral positional dizziness or vertigo were considered to be the diagnostic criteria for pc-BPPV. If only nystagmus was considered, specificity was absolute relative to the sDH, but sensitivity decreased to 50%, which rendered the APCCAM relatively useless. However, considering triggered symptomatology alone to be sufficient for establishing a diagnosis may appear to be a leap of faith in the context of subjective BPPV, an entity that is still debated by clinicians (23, 24). We believe otherwise and suggest that our data strongly support the validity of the concept of subjective BPPV or at least the value of triggered symptomatology alone as being sufficiently reliable for obtaining a diagnosis of BPPV *via* the APCCAM.

Among the 31 patients who presented with only symptoms and no nystagmus after the APCCAM, 30 presented with nystagmus when we conducted the sDH. The remaining patient reported symptoms on the same side when the APCCAM was performed as when the sDH was performed. We considered this patient to have subjective pc-BPPV on both tests and therefore proceeded with the Epley CRP. The patient reported no further vertigo spells at home during follow-up, and the disease was considered resolved.

Whether the absence of nystagmus on the APCCAM in the symptom-only patients was caused by the lower range of head movements that were associated with the maneuver or by the absence of video-oculography during the eye movement assessment is a question that we did not address in this study. This will therefore be the focus of future research.

**Figure 6:**
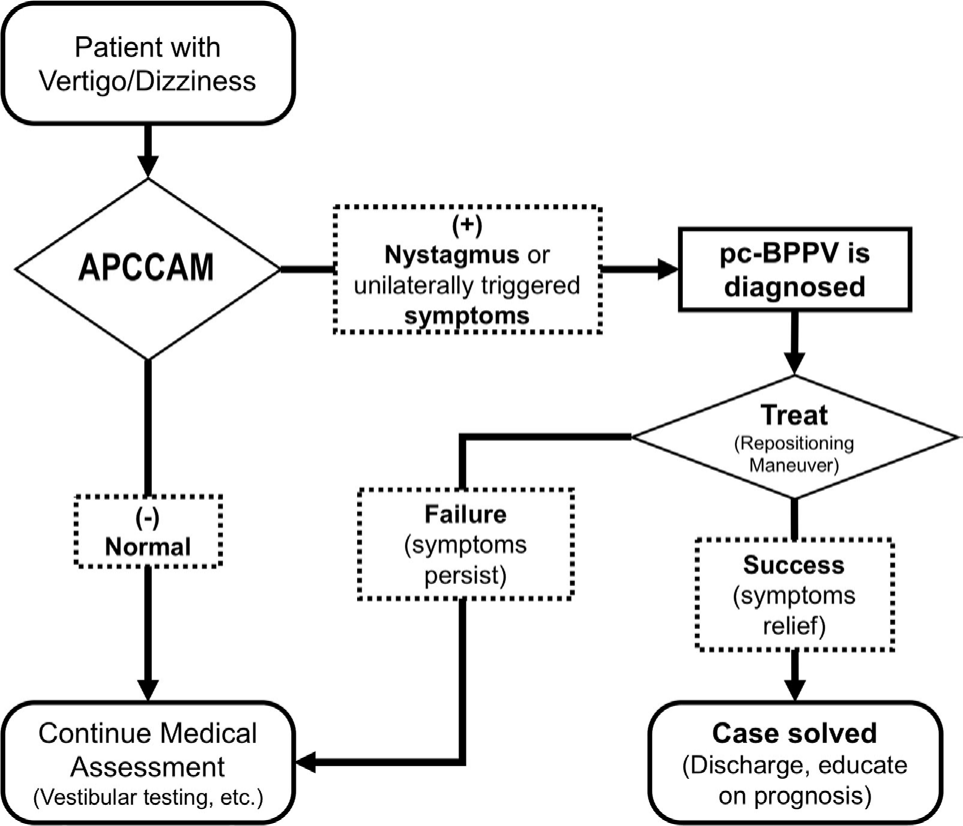
**Conceptual algorithm emphasizing the potential role of the APCCAM**. Because it is the most frequent specific cause of vertigo, identifying pc-BPPV using the APCCAM may lead to instant treatment, thereby avoiding unnecessary referrals or testing.

Importantly, the APCCAM is not intended to replace the sDH for diagnosing BPPV. The role that we suggest for this abbreviated maneuver is illustrated in Figure 6.

Many physicians practicing in primary care or emergency departments, in addition to many otolaryngologists and neurologists, are not properly specialized in vestibular disorders and may find treating patients with vertigo or dizziness to be challenging. The tendency to refer patients and to order routine imaging or vestibular tests varies across different settings. However, as described above, these can be very frequent, which can lead to delays in the diagnosis and treatment of simple cases of BPPV.

Even if we ignored the full range of vestibular disorders, it can take years to acquire the skills and knowledge necessary to manage BPPV and all of its variants. Hence, even experts find some cases to be extremely challenging. However, the maneuver proposed in this study is intended for diagnosing only one variant, and in this context, we believe it will be easy to teach. In support of its use, we have chosen to provide a detailed explanation of the mechanisms and principles that are involved in canal alignment, which underlies the effects of the APCCAM that are described in the introductory segment of this article. We hope that reviewing the pathophysiology of pc-BPPV will improve our understanding and the performance of the APCCAM. This may lead to the implementation of widespread “screening” procedures to diagnose pc-BPPV, and this entity may therefore serve as the first step in an assessment algorithm (Figure 6).

On average, approximately 20% of patients who present with vertigo suffer from BPPV, and 85% of these patients have a pc-BPPV variant. Hence, if APCCAM has an 80% level of sensitivity, we hypothesize that 14% of all vertigo patients would be identified as having pc-BPPV, following the immediate application of the APCCAM upon their first contact with a trained health provider. This could lead directly to CRP. If the patient’s symptoms are completely resolved after treatment, this not only confirms the diagnosis but also ensures that the patient has been properly managed without requiring further tests. However, if the patient’s symptoms persist after a rational number of CRP attempts, the patient should receive an appropriate reassessment.

Because BPPV is associated with other vestibular diseases, the presence of multiple pathologies, including BPPV, may not be uncommon. However, implementing APCCAM would ensure that the BPPV is addressed in these patients. We believe that this algorithm is safe for managing patients with acute vestibular symptoms and no “red flags” that might indicate a severe neurological pathology, such as stroke.

The actual impact of this diagnostic maneuver on patient referral rates, the numbers of tests ordered, and the daily activities of medical centers that treat patients with vertigo, including primary care centers, emergency departments, and vestibular-specialized units, remains to be seen. However, at least in this initial study, the APCCAM appears to be a sensitive and specific tool that can identify the leading and most easily solvable cause of vertigo, BPPV, using a maneuver that can be easily taught and that requires only a backed chair.

## Author Contributions

All authors contributed significantly to the development of the “mini Dix–Hallpike” maneuver presented in this manuscript. All authors contributed to patient assessment and data retrieval. MN, CB, and JF had a key role in vestibular testing and in correcting the manuscript. PM and CO had a key role in the general coordination of the whole project and in statistical analysis (particularly CO). PM and HB were officially responsible for the project. HB and PM wrote the manuscript. All authors participated actively in the corrections and further development of the manuscript.

## Conflict of Interest Statement

The authors declare that the research was conducted in the absence of any commercial or financial relationships that could be construed as a potential conflict of interest.
